# Conversations upon the Physical and Mental Hygiene of Girlhood

**Published:** 1881-08-20

**Authors:** T. S. Powell

**Affiliations:** Atlanta; Professor of Obstetrics and Diseases of Women, and Lecturer on Medical Ethics in the Southern Medical College


### Southern Medical Record:

EDITORS:

T. S. Powell, M.D. W. T. Goldsmith, M.D. R. C. Word, M.D.

*R. C. WORD, M.D., Managing Editor.*

☞All Communications and Letters on Business connected with the Record must be addressed to the Managing Editor.

Vol. XI. ATLANTA, GA., AUGUST 20, 1881. No. 8.

### ORIGINAL AND SELECTED ARTICLES.

### CONVERSATIONS UPON THE PHYSICAL AND MENTAL HYGIENE OF GIRLHOOD.

BY T. S. POWELL, M. D.,

Professor of Obstetrics and Diseases of Women, and Lecturer on Medical Ethics in the Southern Medical College.

### CONVERSATION VIII.

Mother—"Doctor, I was sorry you had to leave so abruptly at your last visit—just in the midst of that interesting subject—physical recreation *versus* brilliant accomplishments, was it not?"

Doctor—"Yes, madam, and here is a paragraph from Canon Kingsley upon the subject of physical education. He says: 'When instructors teach girls not merely to understand the Greek tongue, but to copy somewhat of the Greek training, of that music and gymnastic, which helped to make the cleverest of the old world the ablest race likewise, they will earn the gratitude of the patriot and physiologist, by doing their best to stay the downward tendencies of the physique, and therefore ultimately of the *morals* of the coming generation of women.'"

Mother—"Doctor, you think, then, that one's physical condition has much to do with the character of the person?"

Doctor—"It certainly has, madam. The health of the body has much influence upon the emotional nature or the disposition, and the latter has a great deal to do with the moulding of character. Persons who are perfectly sound in body seldom, if ever, commit a crime. This philosophical and sympathetic relation of the body to the emotional character was understood to some extent in England and France many years ago by some intelligent persons who made no profession of medical attainments, but I regret to know that in our own country at this late day, many of our physicians have but little knowledge of this feature in the economics of medicine, and give no attention to its study."

Mother—"Well, I think they ought to give it a great deal of attention, when, as you say, it has such an important significance among high attainments in their profession."

Doctor—"In the one question of digestion, Sydney Smith said, many years ago, that it was the great secret of life, and that character, talents, virtues and qualities are powerfully affected by beef, mutton, pie crust, and rich soups. He further remarked that he had often thought that he could feed or starve men into many virtues, and affect them more successfully with the instruments of cookery, than Timotheus could formerly do with his lyre."

Mother—"He did, indeed, consider a perfect digestion of great importance."

Doctor—"Now, when a girl or woman is in ill health, has one or more organs diseased, the muscles all enfeebled, her nerves shattered, and consequently a general depression of mind and body, nothing but the aid of a Divine power can enable that poor creature to be cheerful, vivacious and sweet-tempered, if only in a moderate degree; and even this assistance will sometimes fail, because in such a state of health the nerves are actually beyond the invalid's control to a very great extent, and she is irresponsible for their action. By a physiological law, a healthy state of the nervous organism is the very basis of elasticity of the mind and the disposition, and without.it, how can a person be cheerful, pleasant and patient at all times?"

Mother—"Since you mention this, Doctor, I remember how often I have seen corresponding instances, and sometimes in myself, when ill, and also in my daughter Mary, when her health was failing."

Doctor—"Very true, madam, and children, and also adults, are very often rebuked for being impatient and ill-tempered when they really cannot help it. It is cruel as well as unwise to do this, and will retard the recovery of the patient."

Mother—"Then, how shall we do, Doctor, when attending as nurses upon such cases?"

Doctor—"In the first place, have a sympatheticand intelligent conviction that no sick person can possibly feel, in mind or body, like one in good health, and of course, cannot act as one would when in that normal condition. Let the patient see and feel that you have this sympathy and knowledge, and instead of harshly reproving the impatience, irritable temper, and sometimes hysterical burst of tears and fault-finding, try to soothe the poor, quivering nerves with gentle kindness, and after the paroxysm is over talk with the patient affectionately, and tell her that while you know she cannot avoid having those feelings in a great measure, yet to try as much as possible to be quiet and cheerful, since this will do much towards her recovery."

Mother—"I have often noticed that when sick persons were scolded for being irritable, it increased their excitement and frequently ended in hysterical sobs that seemed to exhaust the strength of the patient."

Doctor—"Of course, and augmented her suffering by unnecessary excitation of the already quivering nerves. I repeat, it is unfeeling, and is folly to scold a sick person when in this condition; and, not only every physician and professional nurse should know this, but it ought to be taught the members of every family."

Mother—"Yes, indeed, and I think, Doctor, that the impatience and ill-temper on the part of the patient could be much avoided, perhaps, altogether, if they received proper attention, and everything about them was kept bright and cheerful and pleasantly quiet."

Doctor—"It certainly could, to a great extent, and the whims and caprices of a patient can be indulged without allowing the indulgence to go so far as to really be injurious."

Mother—"I know it requires some one with a kind, feeling heart, and with much patience and intelligence to properly meet all these difficulties when attending the sick."

Doctor—"Yes, it does, madam, but when acting in the capacity of a sick nurse we should all adopt these measures, because it is our duty, and because we like to be treated in this manner when we are ill ourselves."

Mother—"On the principle, I suppose, that the golden rule is an excellent one at the bedside of sick persons as well as everywhere else."

Doctor—"It certainly is, especially in cases like your daughter Mary's, and in every instance when young girls are approaching the age of puberty, whether they are in good health or otherwise, they should be treated with much kindness and affection. At this time the nervous system of a girl is peculiarly sensitive, and to speak to her harshly, or excite her to anger, will cause her much mental suffering, demoralize the amiability of her disposition, and seriously interfere with a vigorous, healthy change from girlhood to the condition of a woman."

Mother—"I have read, somewhere, that when one's body is in a nervous, unhealthy state it acted as a constant irritant and flame to the mind, and of course, every harsh or unkind word adds fuel to the fire."

Doctor—"Very true, madam, but take a girl or woman who has had a perfect physical training, who has been accustomed from infancy to sleep in cool, well ventilated rooms, to eat simple, plain food, exercise much in health-giving work and recreation, drink pure, cold water, and bathe in it daily, practice temperate, prudent, regular habits in all things; and who has been taught the laws of health and life, and how to obey them, so she would know what was demanded of her health and strength, and she is, per force, amiable, sweet-tempered, difficult to vex or excite to anger."

Mother—"But, ah! Doctor, how few we find who are difficult to excite to anger! I have seen young girls get into a passion because a piece of ribbon bought for them was not the exact shade, or when a dress-skirt was an inch too short or too long."

Doctor—"It is not surprising, madam, when we consider that if a girl's physical education has been deficient, that of her morals will be of the same character. But take a girl or woman that has had a proper physical training, as I have just mentioned, and you will find that this education gives her, besides amiability of temper, cheerfulness, vivacity, beauty, vigor of mind as well as body, warmth of heart, and also *moral* stability, more surely than could have been done under opposite conditions."

Mother—"It is intensely interesting to me, Doctor, to learn how much is involved in the training of children, especially our girls, and yet the responsibility is so great I almost shrink from the duty."

Doctor—"Yes, madam, but while that is true, the very importance of the duty ought to make the faithful discharge of it dear to every mother's heart. As you say, the subject is intensely interesting. I know of none more so in the whole range of science, and while the physical training of our children, which by the way should be kept up until they are matured and sufficiently informed to continue it themselves, while this training involves so much responsibility, it is a most delightful duty. It is a beautiful, and even a sublime work to be a co-laborer with the Creator in making his human handiwork perfect in physical, moral and mental development."

Mother—"But, Doctor, is it indeed in our power to do this?"

Doctor—"It is, madam, In a former conversation I think I told you that the mother, aided by the father, can have and should have the ability to give their offspring, both as children and as men and women, perfect development in all these things. It will be more surely accomplished if the education begins before birth. When the mother and father are feeble in health, the child will be very often physically unsound. If they are subject to anger, malice, strife and other evil passions, the child will also partake of their nature, and when the mother never lifts her mind to anything higher than social backbiting, sensational novels or the follies of dress and fashion, and the father is fond of coarse and evil associations, the child will have the same low taste, and small mental and moral power."

Mother—"Such results do seem perfectly natural, and upon philosophic principles."

Doctor—"Yes, madam, and the demonstration can be followed out indefinitely, but it is a solemn truth we cannot get rid of, that both parents, and more especially the mother, make these children, these young men and women we see on the streets and elsewhere ; they mold them as surely as the potter does the piece of clay in his hands. If you could see the perfection of a human being in both father and mother, you would also find it in their children."

Mother—"Oh ! Doctor, I see so much more now than I ever have before and so clearly upon this all important subject. I think you are right in urging and reiterating your advice and suggestions. I have no doubt you find in your practice many cases of deficient training in the health of girls."

Doctor—"I do indeed, madam, some of them very sad, and all could have been avoided by the necessary intelligence on the part of parents and teachers, and proper attention to the health of girls. I had one case of a young girl with curvature of the spine, caused by running up four flights of steps every day to reach the room where she attended school. Another one, approaching the age of puberty, had not sufficient blood and muscular power to successfully effect the change. I advised her father, even urged it upon him, to take her from school for twelve months at least, and to use every hygienic measure as regards exercise, recreation and all others that would give his young daughter more vitality and robustness of health, and warned him if he did not, he might expect distressing, if not fatal results."

Mother—"And did he not take your advice ?"

Doctor—"No, madam, I was only laughed at for my pains. The young girl was kept on in the same way, body and mind overtaxed, became more feeble every day, and finally died, not because the disease itself was necessarily fatal, but because the patient did not have sufficient vitality to overcome an ordinary fever. I, as attending physician, was held responsible for her death."

Mother—"That was very unjust and unkind of the parents, after they had disregarded your advice."

Doctor—"In this case, the mother would have taken my advice if the father had allowed her to do so before it was too late. So you see the obligation rests upon both parents, as I have said, in regard to the health of their daughters."

Mother—"I see that it does. But, Doctor, I and nearly all mothers are delinquent in talking to our daughters at the proper time, so as to impress them with the vital importance of everything that pertains to their health. I would be glad if you would tell me how I shall talk to them about these things."

Doctor—"Yes, madam, I know it is a troublesome subject to mothers, but I do not see why they should have more disinclination to advise their daughters upon this very important subject, than they have to talk to them about the cultivation of their personal appearance and the fashion of a dress. In the first place mothers should divest the subject of their daughters' health, and also that of their boys, of all *false modesty*; until they do this their children will have an idea there is something indelicate about the cultivation of their health, and will therefore think some things that pertain to it must be concealed from their parents.

Mother—"And will perhaps talk about it improperly among themselves."

Doctor—"Certainly, madam, and children of nearly all ages will also get this impression of indelicacy in regard to their health, from hearing the subject spoken of by persons who will treat it coarsely and roughly. Boys will hear it upon the streets, or in other places, from dissolute men or vicious boys, and it will be handled in the presence of girls by some immodest and flippant schoolmate or indiscreet woman, in a manner that will divest the subject of its purity, delicacy, and, shall I say, sacredness?"

Mother—"I think so, Doctor, for when looked at properly, the purity and delicacy of the subject should give it an air of sacredness."

Doctor—"This coarse or indiscreet manner of treating it will have a bad effect upon the morals of a boy or girl, and to the latter especially, if she is timid and sensitive, it will do injury both in mind and health by unduly exciting her imagination and investing this change in her life with a kind of horror, and with fears that are groundless."

Mother—"Yes, I know this from my own experience as a young girl; I was for some time very ignorant about these things, and I had these same fears and imaginary horrors."

Doctor—"And all girls will have them if it is not avoided by the proper instruction and watchfulness of the mother, or some kind and intelligent lady friend. As I have tried to show you, my dear madam, not only an important physical question is here involved, but also a moral one of much significance in the training of our young people. While the mother is giving her young daughter all this necessary information, the father, in the same delicate and reverent manner, should teach his little boys the origin of their being; tell them what their mother suffered to give them life, and what a sacred thing that life is in the sight of the Creator, and should be held so by them, and be preserved in perfect health and vigor. The father would thus quiet in the mind of his boys the anxiety which they all will have in regard to the mystery of life, and also take away the vulgar curiosity in regard to its solution. But, better still, the boys would then grow up with a sympathy, respect and affection for their mother they would not have under other circumstances."

Mother—"I never thought of this before, Doctor, and I can now see plainly which is the better way."

Doctor—"Yes, madam, and as I have said, if the father neglects this duty his sons will learn these things from evil men and boys, and the subject will then, in their minds, be invested with indelicacy or coarseness, and even vulgarity; all of which will surely have a demoralizing effect upon the emotions and the life of his boys."

Mother—"I know that is true, Doctor, and I have often been troubled to think of my children getting such information, and among such associations."

Doctor—"Well, madam, as you see, it can be avoided if the parents will forestall all others by talking to their children themselves. This instruction should be imparted very early. Children comprehend plain and practical lessons in philosophy at a much earlier age than one would suppose. Begin by instructing them in the simplest laws of life and health, and make it an every-day business, as they are growing up, to learn from your teachings, the proper books and practical tests, how to acquire and preserve perfect health of body, mind and morals. Let them learn what kind of food and drink, style of dress, habits of life, in sleeping, exercise, work, and study, reading and recreation will enable them to attain perfection, physically, morally and mentally, and live in happiness and robust health to extreme old age."

Mother—"I have heard some mothers say that a girl ought not to know so much about herself and the disorders and sufferings to which her sex are peculiarly subject, because she will then imagine all the time that she herself is a victim to some of these diseases."

Doctor—"That is a great mistake, madam. On the contrary, when a woman has this intelligent knowledge of herself, she also knows that it is in her power to avoid these diseases, and that conviction itself will give her confidence and courage, and prevent her from being subject to such imaginary conditions."

Mother—"Yes, I understand, and see that your argument is very good."

Doctor—"It is neither necessary nor prudent to give your daughter all this information as a child or a very young girl. After learning the simple rules of health, then when the daughter arrives at the age of twelve or thirteen years, the mother should tell her, in the proper manner, about the change that probably will soon take place in her condition, and impress it upon her to have no fears about it, that it is a natural law with all her sex necessary to her good health; and also, to the perfecting of her beauty in face and form, and that so long as she observes the proper habits of life in every respect, there is no danger at all in this important period of her girlhood. Then, as she grows to woman's maturity, let her learn to know herself *as a woman*, and be well fitted to take care of her health and prevent disease."

Mother—"But, Doctor, does not this change sometimes take place in our own climate before twelve or thirteen years?"

Doctor—"Yes, madam, unfortunately it does, but it is owing to the coarse or luxurious manner in which the child has been reared, more than to difference in temperament, and I do not think that in our country this forced maturity ever argues well for the perfect health and long l fe of the girl after she becomes a woman."

Mother—"Do you not think, Doctor, that the emotional nature of girls undergoes a great change when they first enter their teens?"

Doctor—"It certainly does, madam, and one that especially requires sympathetic and watchful care, until the daughter is at least sixteen years of age. You can probably remember yourself the change that then took place in your mental and emotional nature, as well as in your physical condition. A girl at this period of her life becomes very susceptible to all impressions and emotions, and her nervous system is tremulous and very sensitive. She has feelings, ideas and freaks of imagination she never had before, and which often trouble, and even startle her with the strangeness of their nature. They are a mystery to her, and she thinks and broods over them, and the stamp of her physical and moral being is now perhaps decided for life by the proper information and counsel."

Mother—"She also becomes more excitable and irritable in temper, does she not?"

Doctor—"Yes, madam, and grieves over what she thinks is a lack of amiability, when it is really a physical infirmity she cannot control. Under these conditions the young girl is fortunate if she has a mother who has always had her child's confidence and reliance upon her guidance, and to whom the daughter will intrust all these feelings and have them explained and all her anxiety in regard to them removed from her mind."

Mother—"I can call to mind several lady acquaintances whose life has been most miserable for the want of all these counsels at the proper time. If young girls do not have advisers of their own sex, and older and wiser than themselves, they are in great danger of forming early attachments which end in most unhappy marriages, and they live and die in this miserable condition, throwing all the responsibility upon their mothers. I have heard them say on their death beds: 'Oh! if my mother had only told me all these things, or had encouraged me to talk to her, my life and health would not have been ruined as they now are.'"

Doctor—"Yes, madam, I have known similar cases, and it is all very sad, very distressing. In my professional life I have often discovered the pain in the head, hysterical convulsions and nervous cough of young girls were caused by mental emotion which had been badly directed and indulged in, because it was all concealed from the mother as she did not have the full confidence of her daughter."

Mother—"Doctor, how shall we get this confidence of our children so that they will have no secets of any kind from their parents."

Doctor—"The question you ask is a highly important one—too important for me to attempt to answer now, as my time is out. I will call again in a few days and give you my views. Good morning, madam."

